# Natriuretic Peptides in Post-mortem Brain Tissue and Cerebrospinal Fluid of Non-demented Humans and Alzheimer’s Disease Patients

**DOI:** 10.3389/fnins.2018.00864

**Published:** 2018-11-26

**Authors:** Simin Mahinrad, Marjolein Bulk, Isabelle van der Velpen, Ahmed Mahfouz, Willeke van Roon-Mom, Neal Fedarko, Sevil Yasar, Behnam Sabayan, Diana van Heemst, Louise van der Weerd

**Affiliations:** ^1^Department of Internal Medicine, Section Gerontology and Geriatrics, Leiden University Medical Center, Leiden, Netherlands; ^2^Department of Neurology, Feinberg School of Medicine, Northwestern University, Chicago, IL, United States; ^3^Department of Radiology, Leiden University Medical Center, Leiden, Netherlands; ^4^Department of Human Genetics, Leiden University Medical Center, Leiden, Netherlands; ^5^Percuros BV, Leiden, Netherlands; ^6^Leiden Computational Biology Center, Leiden University Medical Center, Leiden, Netherlands; ^7^Delft Bioinformatics Lab, Delft University of Technology, Delft, Netherlands; ^8^Clinical Research Core Laboratory, Johns Hopkins Institute for Clinical and Translational Research, Baltimore, MD, United States; ^9^Department of Medicine, School of Medicine, Johns Hopkins University, Baltimore, MD, United States

**Keywords:** natriuretic peptides, brain, cerebrospinal fluid, Alzheimer disease, humans, gene expression

## Abstract

Animal studies suggest the involvement of natriuretic peptides (NP) in several brain functions that are known to be disturbed during Alzheimer’s disease (AD). However, it remains unclear whether such findings extend to humans. In this study, we aimed to: (1) map the gene expression and localization of NP and their receptors (NPR) in human post-mortem brain tissue; (2) compare the relative amounts of NP and NPR between the brain tissue of AD patients and non-demented controls, and (3) compare the relative amounts of NP between the cerebrospinal fluid (CSF) of AD patients and non-demented controls. Using the publicly available Allen Human Brain Atlas dataset, we mapped the gene expression of NP and NPR in healthy humans. Using immunohistochemistry, we visualized the localization of NP and NPR in the frontal cortex of AD patients (*n* = 10, mean age 85.8 ± 6.2 years) and non-demented controls (mean age = 80.2 ± 9.1 years). Using Western blotting and ELISA, we quantified the relative amounts of NP and NPR in the brain tissue and CSF of these AD patients and non-demented controls. Our results showed that NP and NPR genes were ubiquitously expressed throughout the brain in healthy humans. NP and NPR were present in various cellular structures including in neurons, astrocyte-like structures, and cerebral vessels in both AD patients and non-demented controls. Furthermore, we found higher amounts of NPR type-A in the brain of AD patients (*p* = 0.045) and lower amounts of NP type-B in the CSF of AD patients (*p* = 0.029). In conclusion, this study shows the abundance of NP and NPR in the brain of humans suggesting involvement of NP in various brain functions. In addition, our findings suggest alterations of NP levels in the brain of AD patients. The role of NP in the development and progression of AD remains to be elucidated.

## Introduction

**FIGURE 1:**
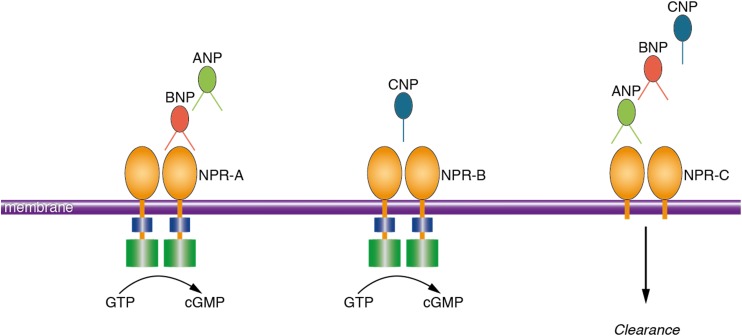
Schematic presentation of the ligand preference of natriuretic peptide receptors. Natriuretic peptides bind to three transmembrane receptors, namely NPR-A, NPR-B, and NPR-C. ANP and BNP bind to NPR-A; CNP binds to NPR-B and all the NP (ANP, BNP, and CNP) bind to NPR-C. Activation of the NPR increases the intracellular concentration of cyclic guanosine monophosphate which is the main mediator of the biological activities of NP. NPR-C is a clearance receptor. ANP, atrial natriuretic peptide; BNP, brain natriuretic peptide; NPR-A, natriuretic peptide receptor A; NPR-C, natriuretic peptide receptor C; GTP, guanosine triphosphate; cGMP, cyclic guanosine monophosphate.

Natriuretic peptides (NP) refer to a group of peptides that are mostly known for their actions within the cardiovascular system (Potter et al., 2006). NP are mainly secreted from cardiac myocytes in response to cardiac wall stretch and volume expansion. Consequently, NP regulate body fluid homeostasis by inducing natriuresis, diuresis, vasodilation and lowering blood pressure (Potter et al., 2006). Three members of this family are atrial natriuretic peptide (ANP), brain natriuretic peptide (BNP), and C-type natriuretic peptide (CNP) (Suzuki et al., 2001). The biological activities of NP are mediated through activation of three transmembrane receptors including natriuretic peptide receptor A (NPR-A), natriuretic peptide receptor B (NPR-B), and natriuretic peptide receptor C (NPR-C) (Potter et al., 2006; Figure 1). While NP were initially discovered in cardiac myocytes (de Bold et al., 1981), extensive distribution of NP and their receptors in the brain of animal species has been repeatedly reported (Cao and Yang, 2008). NP and their receptors were shown to be present in various neuronal structures, glial cells and cerebral vessels (Cao and Yang, 2008; Prado et al., 2010). Notably, findings from animal studies suggest that NP may regulate neuroplasticity (Decker et al., 2010), blood-brain barrier integrity (Bohara et al., 2014), neuro-inflammation (Moriyama et al., 2006; James et al., 2010) and memory function (Telegdy et al., 2000; Decker et al., 2009).

The biological roles of NP in the central nervous system (CNS) have been mainly described in rodent and mammalian species (Mahinrad et al., 2016). Existing evidence from humans indicates that higher plasma levels of NP associate with dementia and accelerated cognitive decline (van der Velpen et al., 2017). Although this link was mainly attributed to cardiovascular pathologies (Wang et al., 2004), recent findings suggest that plasma NP associate with cognitive decline independent of cardiovascular disease (Wijsman et al., 2014; Sabayan et al., 2015). Moreover, higher plasma levels of BNP were linked with lower cerebrospinal fluid (CSF) amyloid beta42 (Aβ42) and total tau/Aβ42 ratios (Hu et al., 2012). Interestingly, some studies have reported the presence of NP and their receptors in brain tissue and CSF of humans (Ogawa et al., 1992; Kaneko et al., 1993). Hence, we have recently hypothesized that NP may be potential diagnostic and/or therapeutic markers for Alzheimer’s disease (AD) (Mahinrad et al., 2016).

To substantiate this hypothesis, in this study we aimed to: (1) map the gene expression and localization of NP and their receptors (NPR) in human post-mortem brain tissue; (2) compare the relative amounts of NP and NPR between the brain tissue of AD patients and non-demented controls, and (3) compare the relative amounts of NP between the CSF of AD patients and controls.

## Materials and Methods

### Experimental Design

We had access to biomaterials of 17 non-demented controls (mean age = 80 ± 8.5 years, 59% female) and 10 patients with a definitive pathological diagnosis of AD (mean age 85.8 ± 6.2 years, 60% female) (Table 1). The definitive diagnosis of the donors was provided by expert neuropathologists that considered both histopathological results and the clinical diagnosis of the donors. To investigate the localization of NP and NPR in the brain, we performed immunohistochemistry using post-mortem brain tissue of a subset of 13 non-demented controls and 10 AD patients (Table 1). For quantitative assessment of NP and NPR in the brain, we performed Western blotting using a subset of donors from whom frozen brain tissue was available (10 controls and 8 AD patients, Table 1). NP in the CSF were detected by enzyme-linked immunosorbent assay (ELISA) in another subset of the donors that had a frozen CSF sample available (10 controls and 10 AD patients, Table 1). Age and gender were not significantly different between controls and AD patients in any of the subsets (all *p* > 0.05).

**Table 1:** Characteristics of study participants.

Diagnosis	Source	Age	Gender	PMD (h)	IHC	WB	CSF	CSF pH
Control	NBB	84	F	5:36				6.68
Control	NBB	70	F	7:35		NA		6.03
Control	NBB	64	F	5:40		NA	NA	
Control	NBB	83	M	5:15		NA		6.60
Control	NBB	91	F	3:47				6.27
Control	NBB	73	M	8:00				5.37
Control	NBB	72	F	6:50				7.22
Control	NBB	89	F	6:30				6.55
Control	NBB	88	M	11:10	NA	NA		5.98
Control	NBB	82	M	5:20	NA	NA		5.85
Control	NBB	73	F	5:30	NA	NA		6.38
Control	NABCA	82	M	5:30		NA	NA	
Control	NABCA	87	F	8:30			NA	
Control	NABCA	72	F	7:15			NA	
Control	NABCA	93	M	8:30			NA	
Control	NABCA	82	M	7:30			NA	
Control	NABCA	73	F	6:30	NA		NA	
AD	NBB	88	M	5:30				6.72
AD	NBB	85	F	4:05				6.47
AD	NBB	86	M	5:10		NA		6.61
AD	NBB	81	M	7:50				6.15
AD	NBB	88	F	4:40				6.39
AD	NBB	73	M	4:45				6.48
AD	NBB	96	F	7:55				5.90
AD	NBB	82	F	4:35		NA		6.53
AD	NBB	90	F	3:55				6.24
AD	NBB	89	F	4:30				6.73

*AD, Alzheimer’s disease; NBB, Netherlands Brain Bank; NABCA, Netherlands Aging Brain Collection Amsterdam; PMD, post-mortem delay (in hours); IHC, immunohistochemistry; WB, Western blot; NA, not available; CSF, cerebrospinal fluid*.

### Brain Tissue and CSF Samples

Post-mortem brain tissue from the frontal lobe – middle frontal gyrus – of 10 AD patients and 8 controls were obtained from the Netherlands Brain Bank (NBB, Netherlands Institute for Neuroscience Amsterdam); and for 6 controls tissues were obtained from the Normal Aging Brain Collection Amsterdam (NABCA). All CSF samples were provided by the NBB. The CSF was collected from the lateral ventricles during autopsy and stored at -80°C for later analysis. All donors gave written informed consent for brain autopsy and for the use of their specimens and medical records for research purposes. According to national ethical guidelines, all samples were coded to maintain the anonymity of donors.

### Gene Expression Data

To investigate the gene expression of NP and NPR in the brain, we used BrainScope (Huisman et al., 2017).^1^ BrainScope is a web-portal for visual analysis of gene expression in the brain of healthy humans, based on data from the Allen Human Brain Atlas (Hawrylycz et al., 2012; Hawrylycz et al., 2015).^2^ The Allen Human Brain Atlas provides the mRNA expression of about 20,000 genes using six healthy adult donors (age range 24 to 57 years). Gene expression was measured with a customized micro-array chip using ∼3,700 samples taken from anatomically annotated regions of the brain. From this data, BrainScope uses 105 expression values per gene corresponding to anatomically annotated brain regions that were sampled for all the six donors. The expression values were averaged to provide a single expression value for each sample across the individuals (Huisman et al., 2017). Accordingly, we used BrainScope to explore the expression of the following genes: natriuretic peptide A (*NPPA*, encodes ANP protein, Gene ID 4878), natriuretic peptide B (*NPPB*, encodes BNP protein, Gene ID 4879), natriuretic peptide C (*NPPC*, encodes CNP protein, Gene ID 4880), natriuretic peptide receptor 1 (*NPR1*, encodes NPR-A protein, Gene ID 4881), natriuretic peptide receptor 2 (*NPR2*, encodes NPR-B protein, Gene ID 4882) and natriuretic peptide receptor 3 (*NPR3*, encodes NPR-C protein, Gene ID 4883).

### Immunohistochemistry

Formalin-fixed, paraffin-embedded tissues were serially cut into 5-μm-thick sections and mounted on coated glass slides (SuperFrost^®^Plus, VWR). The sections were deparaffinized in xylene and rehydrated through graded ethanol concentrations (100, 90, and 70%). To block endogenous peroxidase activity, the sections were incubated for 20 min in methanol with 0.3% hydrogen peroxide (H_2_O_2_). This was followed by antigen retrieval by cooking the sections for 20 min at 0.76 bar steam pressure in an acidic pH 6 solution (H-3300, Vector labs). The antigen retrieval step was performed depending on the primary antibody (Supplementary Table 1). Next, the sections were washed in phosphate-buffered saline (PBS) and incubated with the primary antibody overnight at room temperature in the blocking buffer (1% bovine serum albumin [BSA] in PBS). The sections were then incubated for 1 h with a biotin-labeled secondary anti-rabbit or anti-mouse antibody followed by 30 min incubation with avidin-biotin complex (ABC, Vector Labs, CA, United States). For the anti-NPR-C primary antibody, the secondary antibody was conjugated to horseradish peroxidase(HRP) instead of biotin. Staining was visualized using 3,3′-Diaminobenzidine (DAB) which was activated by H_2_O_2_. Finally, the sections were counterstained with Harris Haematoxylin and coverslipped with Entellan. The sections were scanned using an automatic bright field slide scanner (Philips IntelliSite Ultra Fast Scanner, Digital pathology slide scanner, Netherlands) for microscopic evaluation.

All sections were evaluated for the presence/absence of NP or NPR staining in the gray matter (GM), white matter (WM) and cerebral vessels. The assessments were performed by two trained observers (SM and IV) who were blinded to the clinical diagnosis of the donors. Disagreements (*n* = 151 cases, 9.8% of the sections) between the two reviewers were resolved by discussion with the third reviewer (MB). For semi-quantitative comparisons, we compared the percentage of the positively scored subjects (presence of signal) between AD patients and controls for each protein of interest.

### Protein Isolation and Western Blotting

The frozen brain tissues were chopped in GM and WM on dry ice. We were not able to separate the WM of four AD patients due to severe atrophy of the brain tissue. Next, the GM and WM samples were suspended in lysis buffer (50 mM tris pH 7.5 and 1% triton in 10 ml Milli-Q) supplemented with cOmplete Mini Protease Inhibitor Cocktail Tablets (Roche). This was followed by homogenizing the samples in a bullet blender electric homogenizer (Next Advance) for 3 min using 0.5 mm stainless steel beads. The homogenized samples were centrifuged for 30 min at full speed before collection of the supernatants. Protein concentration was calculated using the bicinchoninic acid kit (Thermo Fisher Scientific, Waltham, MA, United States) with bovine serum albumin as a standard. The supernatant was further diluted in sample buffer and denatured by boiling for 10 min in 95°.

We used 50 μg of the GM and WM protein lysates per sample. Proteins were separated by SDS-PAGE by running through a sodium dodecyl sulfate 4–20% polyacrylamide gradient gel (Bio-Rad) alongside a proteins size marker (PageRuler^TM^, Thermo Fisher Scientific). Proteins were transferred to a nitrocellulose membrane using the *Trans*-blot Turbo Transfer system (Bio-Rad) for 30 min at 1.0 A. The transfer of proteins was checked with Ponceau red followed by washing the membrane in Tris-Buffered Saline (TBS). The membrane was blocked in 5% low fat milk in TBS-Tween (mTBST) for 1 h and incubated with primary antibody (diluted in 5% mTBST) at room temperature for 90 min, 2 h or overnight at -4°, depending on the antibody used. After washing in TBS buffer, the membrane was incubated with secondary antibodies: anti-rabbit or anti-mouse IRDye800CW (LI-COR, Lincoln, NE, United States) at 1:5000 for 1.5 h. For β-actin loading control, the membrane was incubated with mouse β-actin at 1:5000 followed by a secondary anti Mouse IRDye680CW antibody (LI-COR, Lincoln, NE, United States). Target bands were visualized using an Odyssey infrared imaging system (LI-COR). The relative densities of the bands were measured using Image Studio Lite software (version 5.0).

### CSF Analysis Using ELISA

The frozen CSF samples were shipped to Johns Hopkins University, Baltimore, MD, United States, for ELISA experiments. ANP, BNP, and CNP were measured using commercial ELISAs following the manufacturer’s protocol (ALPCO, Salem, NH, United States), except that CSF was concentrated prior to analysis by centrifugation/lyophilisation. For ANP and BNP, samples were reconstituted or diluted in diluent 35 (Mesoscale Diagnostics, Rockville, MD, United States) and for a subset of donors CSF was spiked with a mid-range NP standard and recovery was assessed. A sandwich pro-ANP ELISA (1–98) was used to quantify ANP where CSF samples taken to dryness were resuspended in a volume to generate a 5-fold concentration. The sensitivity of the assay was 0.05 nmol/L and the dynamic range was 0.63 to 10 nmol/L. The amino terminal BNP fragment (nt-pro-BNP 8–29) was measured by competitive ELISA where CSF samples were also concentrated 5-fold. Under the conditions used in the laboratory, the sensitivity was 34 pmol/L and the dynamic range was between 40 and 1600 pmol/L. Determination of levels of amino-terminal pro-CNP was performed using a sandwich ELISA where CSF was diluted 1:30 with diluent 35 prior to analysis. The sensitivity was 0.7 pmol/L and the dynamic range was 0.1 to 128 pmol/L. When 9 CSF samples were spiked with 1.25 nmol/L pro-ANP, the recovery was 87 ± 9%. Nine samples spiked with 400 pmol/L pro-BNP yielded a recovery of 103 ± 5%, while for 9 samples spiked with 20 pmol/L pro-CNP the recovery was 93 ± 5%.

### Statistical Analysis

The semi-quantitative immunohistochemistry scores were compared between AD patients and controls using *x^2^* test. Depending on the distribution of the western blot or CSF measures-, unpaired two-tailed *t*-test or Mann–Whitney *U*-test were used to compare between AD patients and controls. We used Pearson correlation coefficient to assess the effect of postmortem delay on NP or NPR levels in postmortem brain tissue and ventricular CSF. Statistical analyses were performed using SPSS software and a *p*-value < 0.05 was considered as statistically significant.

## Results

### Gene Expression of NP and NPR in the Brain of Healthy Humans

**FIGURE 2:**
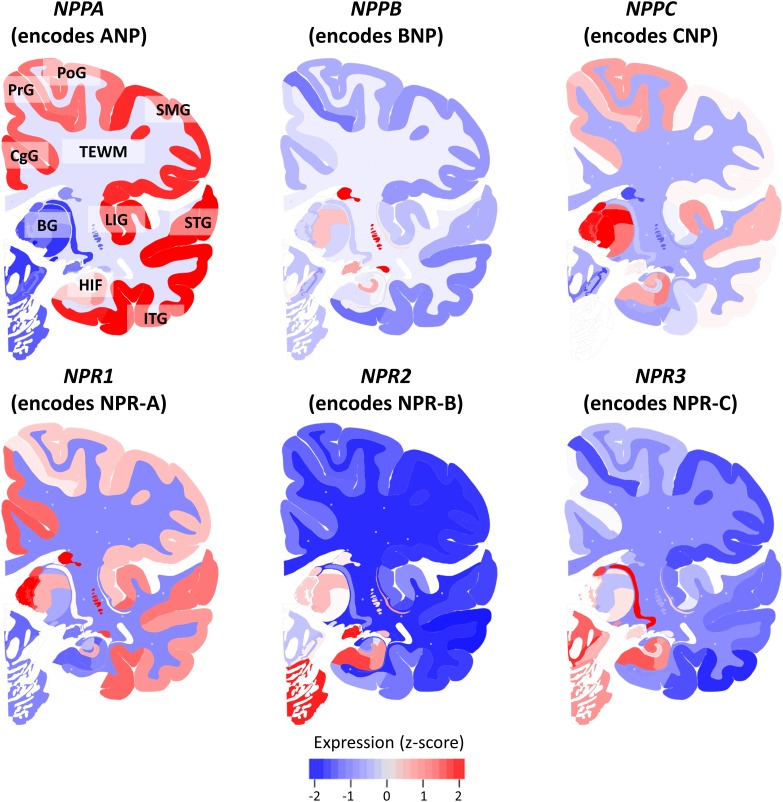
Expression of natriuretic peptides’ and natriuretic peptide receptors’ genes in the healthy human brain. The mRNA expression of genes coding for natriuretic peptides and natriuretic peptide receptors is shown in a coronal view of the left hemisphere. The Expression of each gene is *z*-score normalized to the average expression of the gene in the whole brain. Data from the Allen Human Brain Atlas (http://human.brain-map.org/) is visualized using the BrainScope portal (http://www.brainscope.nl/). CgG: cingulate gyrus; PrG: precentral gyrus; PoG: postcentral gyrus; SMG: supramarginal gyrus; LIG: long insular gyrus; STG: superior temporal gyrus; ITG: inferior temporal gyrus; HIF: hippocampal formation; BG: basal ganglia and TEWM: telencephalic white matter.

Figure 2 shows the mean expression of genes coding for NP and NPR across the brains of six healthy human donors from the Allen Human Brain Atlas. The NP and NPR genes were expressed throughout the CNS, although the different NP had distinctly diverse regional expression levels. In particular, the gene coding for ANP (*NPPA*) had the highest expression in cortical regions and hippocampus, followed by a lower expression in the telencephalic white matter regions (TEWM) and the lowest expression in the subcortical structures. In contrast, the gene coding for BNP (*NPPB*) had the highest expression in subcortical structures (hippocampus and basal ganglia), followed by a lower expression in the TEWM and very low expression throughout the cortical regions. The gene coding for CNP (*NPPC*) had the highest expression in subcortical structures (thalamic nuclei and hippocampus), followed by a lower expression in the cortical regions and the lowest expression in TEWM. The gene coding for NPR-A (*NPR1*) had the highest expression in subcortical structures (basal ganglia and hippocampus), followed by a lower expression in cortical regions and the lowest expression in the TEWM. The genes coding for NPR-B (*NPR2*) and NPR-C (*NPR3*) had the highest expression in subcortical structures (basal ganglia and hippocampus) followed by a very low expression in cortical and TEWM regions.

### Localization of NP and NPR in the Brain Tissue of AD Patients and Controls

**FIGURE 3:**
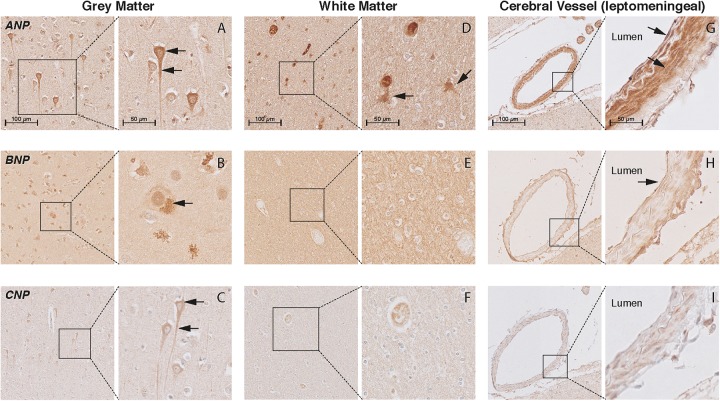
Localization of ANP, BNP, and CNP in the frontal cortex of non-demented controls. NP immunohistochemistry in the frontal lobe (middle frontal gyrus) of non-demented controls. **(A–C)** ANP, BNP, and CNP-positive neurons. Arrows point to the cytoplasm (ANP and CNP), neuronal processes (ANP and CNP), and Nissl bodies (BNP); **(D)** ANP-positive astrocyte-like cells in the white matter; **(E,F)** negative BNP and CNP staining in the white matter; **(G)** ANP-positive endothelium and smooth muscles; **(H)** BNP-positive endothelium, and **(I)** negative CNP staining in the leptomeningeal vessels.

**FIGURE 4:**
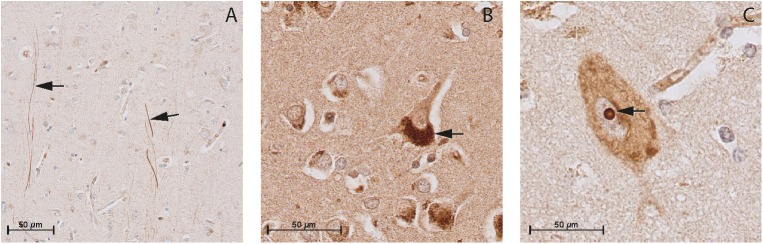
Immunohistochemistry of CNP, NPR-B, and NPR-A in the gray matter of non-demented controls. **(A)** Immunohistochemistry of CNP in the gray matter. Arrows point to CNP-positive fibers in the gray matter; **(B)** Immunohistochemistry of NPR-A in the gray matter. Arrow point to the granular bodies resembling Nissl bodies in the cytoplasm of neuron, and **(C)** Immunohistochemistry of NPR-B in the gray matter. Arrow point to the nucleolus of the neuron.

**FIGURE 5:**
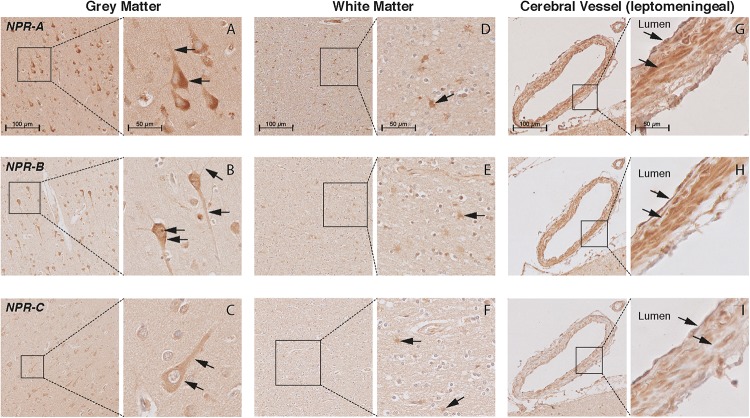
Localization of NPR-A, NPR-B, and NPR-C in the frontal cortex of non-demented controls. NPR immunohistochemistry in the frontal lobe (middle frontal gyrus) of non-demented controls. **(A–C)** NPR-A, NPR-B, and NPR-C-positive neurons. Arrows point to the cytoplasm (all NPR), neuronal processes (all NPR), and Nissl bodies (NPR-A); **(D–F)** NPR-A, NPR-B, and NPR-C-positive astrocyte-like cells in the white matter; **(G–I)** NPR-A, NPR-B, and NPR-C-positive endothelium and smooth muscles in leptomeningeal vessels.

In the cortex of controls, the immunohistochemistry study showed positive staining of all NP and NPR proteins in the neuronal structures. ANP, BNP, and CNP positive stainings were observed in the cytoplasmic body and neuronal processes of pyramidal neurons (layers II–VI) (Figures 3A–C, respectively). BNP-positive staining was mainly localized to Nissl bodies that were characterized as granular bodies within the cytoplasm of neurons (Figure 3B). In addition, the CNP-positive staining was observed in networks of short and long fibers throughout the cortex (Figure 4A). NPR-A, NPR-B, and NPR-C positive stainings were also localized to the cytoplasmic body and neuronal processes of pyramidal neurons (II–VI) (Figures 5A–C, respectively). Furthermore, a strong NPR-A positive staining was observed in the Nissl body of neurons throughout the cortex. These Nissl bodies were characterized as large granular bodies in the cytoplasm with a stronger staining pattern compared to the cytosol of neurons (Figure 4B). In addition to the cytoplasm, the NPR-B-positive neurons also showed a prominent staining in the nucleolus of neurons (Figure 4C). We observed no staining in the negative control sections using only the secondary antibody (Supplementary Figure 1).

In the WM, we observed ANP-positive astrocytes-like structures that consisted of short packed processes (Figure 3D). These astrocytes-like structures were spread throughout the WM, occasionally associated with blood vessels and their cell bodies were not always distinct. Some ANP-positive astrocytes-like structures were also observed throughout the cortical layers. Similarly, we observed NPR-positive astrocyte-like staining in the WM of some controls (Figures 5D–F). We did not observe BNP or CNP-positive astrocyte-like structures in the WM or GM (Figures 3E,F). Other glial cells were not stained for NP or NPR.

In the cerebral vessels, ANP-positive staining was observed in the leptomeningeal and parenchymal vessels, and was localized to the endothelium and smooth muscle layers (Figure 3G). BNP-positive staining was only weakly present in the endothelium of some leptomeningeal vessels (Figure 3H), while CNP staining was not detected in the cerebral vessels at all (Figure 3I). NPR-A and NPR-B stainings were positive in the endothelium and smooth muscle layers of leptomeningeal and parenchymal vessels (Figures 5G,H), while NPR-C staining was less prominent in the cerebral vessels (Figure 5I). Supplementary Figures 2, 3 show the localization of the NP and NPR in the frontal lobe of AD patients. We observed a similar pattern of NP and NPR staining in the frontal lobe of AD patients and controls. Finally, our semi-quantitative results showed a higher percentage of NPR-A-positive astrocytes-like structures in AD patients compared to controls (70% positive staining in AD patients vs 23% positive staining in controls, *x*^2^= 5.07, *df* = 1, *p* = 0.024). The staining in other structures including neurons and leptomeningeal vessels were not significantly different between AD patients and controls (Supplementary Table 2).

### Quantitative Comparison of NP and NPR in Brain Tissue Between AD Patients and Controls

**FIGURE 6:**
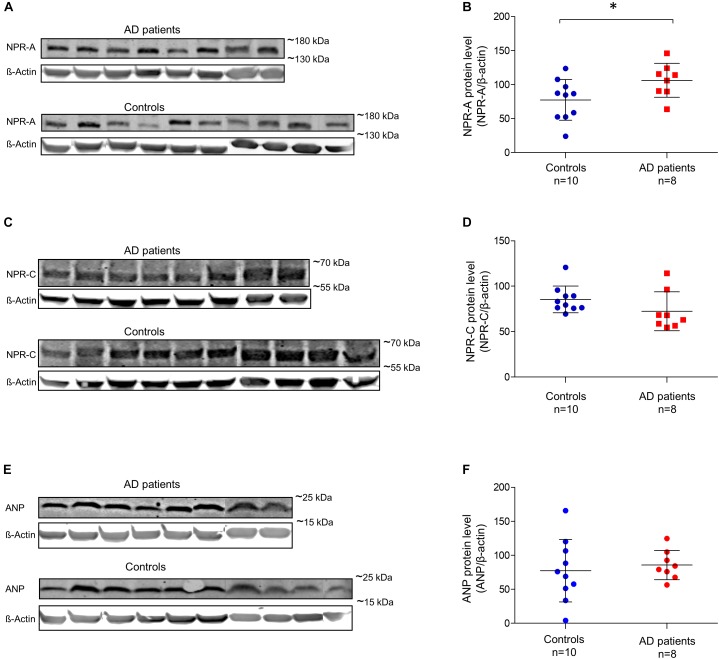
Relative amounts of ANP, NPR-A, and NPR-C in the gray matter of non-demented controls and Alzheimer’s disease patients. **(A)** NPR-A and β-Actin Western blot bands in AD patients and non-demented controls; **(B)** quantification of NPR-A Western blots between AD patients and controls; **(C)** NPR-C and β-Actin Western blot bands in AD patients and non-demented controls; **(D)** quantification of NPR-C Western blots between AD patients and controls; **(E)** ANP and β-Actin Western blot bands in AD patients and non-demented controls, and **(F)** quantification of ANP Western blots between AD patients and controls. Bars show the mean (standard deviation) of the normalized signals. Analyses were performed using *t*-test for NPR-A and Mann-Whitney *U*-test for NPR-C and ANP. ^∗^*p* < 0.05. ANP, atrial natriuretic peptide; NPR-A, natriuretic peptide receptor A; NPR-C, natriuretic peptide receptor C; AD, Alzheimer’s disease.

For quantitative comparisons in brain tissue, we performed Western blots using GM and WM samples isolated from each donor. Using an antibody that binds to ANP, we observed an expected band of ∼17 kDa in both AD patients and controls (Supplementary Figure 4). ANP was abundant in the GM, but virtually absent in the WM. Using an antibody against NPR-A, we observed specific bands of ∼150 kDa in both AD patients and controls. The NPR-A specific bands were highly pronounced in the GM while no or very weak bands were present in the WM (Supplementary Figure 4). Bands reacting with anti-NPR-C antibody were observed at ∼60 kDa in both AD patients and controls; and in GM and WM samples (Supplementary Figure 4). We were not able to detect any BNP, CNP, or NPR-B specific signals despite the use of different antibodies. Figure 6 shows quantification of Western blot bands in the GM of AD patients and controls. We found significantly higher amounts of NPR-A in the GM of AD patients compared to controls [*t*(16) = -2.18, *p* = 0.045, independent sample *t*-test]. NPR-C signals appeared to be higher in the GM of controls, although the differences between AD patients and controls were not statistically different (Mann–Whitney *U* = 18.0, *p* = 0.051 two-tailed). Similarly, ANP signals were not significantly different between AD patients and controls (Mann–Whitney *U* = 33.0, *p* = 0.534). In the WM, none of the Western blot bands was significantly different between AD patients and controls (data not shown). Finally, there was no significant relationship between the postmortem delay and ANP, NPR-A or NPR-C levels in the brain tissue (ANP *r* = -0.45, NPR-A *r* = -0.21, and NPR-C *r* = 0.38; all *p*-values > 0.05).

When comparing between GM and WM, ANP, and NPR-A-specific signals were significantly higher in GM compared to WM (*p* < 0.001), while NPR-C-specific signals were not different between GM and WM. Comparison of the gene expression data (Figure 2) with our Western blot findings (Figure 6) showed similar patterns: the genes coding for ANP and NPR-A were highly expressed in the cortex (expression *z*-score > 0) compared to the TEWM (expression *z*-score < -1). On the other hand, the gene coding for NPR-C had low mRNA expression in both GM and WM (expression *z*-score < 0 in GM and WM).

### Quantitative Comparison of NP in the CSF Between AD Patients and Controls

**FIGURE 7:**
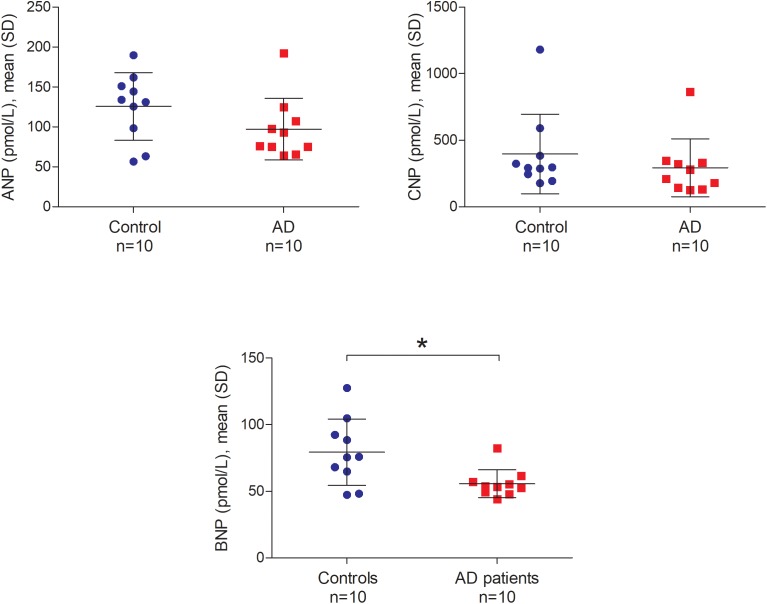
ANP, BNP, and CNP in the post-mortem cerebrospinal fluid of non-demented controls and Alzheimer’s disease patients. Bars represent the mean (standard deviation) of each NP in the cerebrospinal fluid of AD patients and controls. Analyses were performed using Mann–Whitney U test. ^∗^*p* < 0.05. CSF, cerebrospinal fluid; AD, Alzheimer’s disease; ANP, atrial natriuretic peptide; BNP, brain natriuretic peptide; CNP, C-type natriuretic peptide; SD, standard deviation.

Atrial natriuretic peptide (ANP), BNP, and CNP levels in the CSF ranged from 56.48 to 192.36 pmol/L, 44.12 to 127.39 pmol/L and 125.25 to 1180.13 pmol/L, respectively. We did not find any significant relationship between postmortem delay and NP levels in the CSF (ANP *r* = -0.04, BNP *r* = 0.22 and CNP *r* = 0.17; all *p*-values > 0.05). Furthermore, there was no relationship between CSF pH and NP levels in the CSF (ANP *r* = -0.05, BNP *r* = -0.07 and CNP *r* = -0.17; all *p*-values > 0.05). Figure 7 shows the comparison of ANP, BNP and CNP levels between AD patients and controls in post-mortem CSF. We observed significantly lower amounts of BNP in the CSF of AD patients compared to controls (Mann–Whitney *U* = 21.0, *p* = 0.029 two-tailed). Similarly, ANP and CNP levels appeared to be lower in the CSF of AD patients although these differences were not statistically significant (Figure 7).

## Discussion

In this study, we first showed that the genes coding for NP and NPR are ubiquitously expressed across the brain of healthy humans. We detected NP and NPR proteins in neuronal structures, cerebral vessels, and in structures resembling astrocytes in the frontal lobe of non-demented individuals and patients with AD. When comparing between AD patients and non-demented controls, NPR-A levels were higher in the brain tissue of AD patients while BNP levels were lower in the CSF of AD patients.

Previous animal studies have detected the expression and localization of NP and NPR in numerous structures of the CNS (Mahinrad et al., 2016). For example, ANP was detected in neuronal structures of the hypothalamus, telencephalon, and cerebellum (Cao and Yang, 2008), and in the astrocytes of human and canine cortex (McKenzie, 1992; McKenzie et al., 1994). Similarly, BNP and CNP were found to be widely distributed in the neuronal structures of the cerebral cortex, hypothalamus, spinal cord, and retina (Cao and Yang, 2008; Mahinrad et al., 2016). Furthermore, the receptors for ANP and BNP were detected on the luminal membrane of cerebral vessels *in vitro* and *in vivo* (Ermisch et al., 1991; Gelfand et al., 1991). Despite the wealth of evidence from animal studies, limited data on the distribution and expression of NP in the brain of humans is available. In this study, we extended the evidence from animal species to human by providing a detailed mapping of NP and NPR in the frontal lobe of humans. As in animal species, in humans the expression of NP and NPR genes seems to be complementary in various brain regions (Cao and Yang, 2008). This suggests that NP are not only produced in cardiac myocytes, but are also locally produced in the CNS of humans (Cao and Yang, 2008). Furthermore, we found that NP and NPR are present in various cellular structures of the frontal lobe, suggesting a wide range of NP functions in the human brain. In particular, the presence of NP and NPR in both neuronal and cerebral vessels might indicate a potential role of NP in neurovascular functioning (Iadecola, 2004). In line with this, recent *in vitro* studies have shown that administration of ANP and BNP resulted in a significant dose-dependent relaxation of the middle cerebral artery and basilar artery (Guo et al., 2015). On the other hand, NP might also be involved in synaptic transmission and processing of information. In support of this, animal and *in vivo* studies have demonstrated that NP regulate the release and re-uptake of neurotransmitters such as noradrenalin, dopamine and glycine (Mahinrad et al., 2016). We could also detect ANP and NPR in structures resembling astrocytes in the WM. Specifically, we found that NPR-A positive astrocytes-like cells were more frequently observed in AD patients compared to controls. This is consistent with previous studies showing the presence of ANP and NPR in astrocyte of the cortex (McKenzie, 1992), suggesting a potential role of astrocytes in physiological functions of NP in the brain (Mahinrad et al., 2016). Further experiments using specific antibodies against astroglia cells will verify the identity of these astrocyte-like structure. Collectively, our qualitative and gene expression data are consistent with previous animal studies indicating NP as neuropeptides that might regulate several functions in the brain (Hodes and Lichtstein, 2014). Nevertheless, future research is needed to further disentangle the putative role(s) of centrally acting NP in the brain of humans.

Given the involvement of NP in several of the pathological pathways that are also disturbed during AD, we have recently hypothesized NP as potential markers for diagnosis and/or treatment of AD (Mahinrad et al., 2016). We postulated that decreased action of NP in the brain might impair the structural and/or functional integrity of the brain and predispose individuals to a higher risk of cognitive decline (Mahinrad et al., 2016). In line with this hypothesis, we found lower levels of BNP in the CSF of AD patients, coupled with higher amounts of NPR-A in the brain tissue of AD patients. This may suggest impaired function of NP in the brain of AD patients, which could in turn accelerate neuro-inflammation, oxidative stress and neurodegeneration. Such alteration may ultimately hamper the functional and/or structural integrity of the brain and predispose individuals to a higher risk of cognitive decline. One explanation for reduced levels of BNP in the CSF could be attributed to their elevated levels in the systemic circulation. In fact, systemic and central NP might act in a feedback loop such that increased NP in the plasma inhibits production and/or biological activity of NP in the brain (Pemberton et al., 2002; Mahinrad et al., 2016). In line with this, previous research has shown that higher levels of BNP in the plasma of ovine sheep associates with decreased BNP levels in the hypothalamus (Pemberton et al., 2002). Interestingly, recent findings in humans suggest that higher plasma BNP associates with lower CSF Aβ42 and higher *t*-tau/Aβ42 ratios (Hu et al., 2012).Collectively, our pilot results point toward a potential role of NP in the pathology of AD. Nevertheless, larger scale studies are needed to replicate our findings and explore the potential association of centrally acting NP with markers of neurodegeneration. In addition, it should be noted that our results cannot directly be extrapolated to lumbar CSF since we used ventricular CSF in our experiments. In this setting, future research should determine the potential differences in NP levels between ventricular and lumbar CSF. Furthermore, it is not known whether post-mortem delays affect the quantification of NP in the brain. In this study, we did not find a significant correlation between NP levels and postmortem delay, but more detailed studies are needed to further assess this potential confounding effect. Finally, the effect of cardiovascular risk factors, cardiovascular co-morbidities and use of medications on NP in the brain should be addressed in future.

We used frontal lobe to detect NP and NPR in the brain tissue of AD patients and non-demented controls. Although hippocampus is the primary brain region being affected during AD, frontal lobe involvement is also a well-described feature of AD pathology (Laakso et al., 1995; Lehtovirta et al., 1995; Du et al., 2007). Furthermore, atrophy of the middle frontal gyrus has been demonstrated in patients with mild cognitive impairment (Apostolova and Thompson, 2008). It is worth mentioning that previous research in animals have detected NP and NPR in various brain regions such as hippocampus, thalamus and hypothalamus (Cao and Yang, 2008). Consistent with this, we observed high expression of NP and NPR in the subcortical structures including hippocampus and hypothalamus in healthy humans. In this setting, future studies focusing on other brain regions are needed to further disentangle the localization and function of NP in different brain regions of humans.

In summary, we showed the widespread presence of all NP and their receptors in the brain of humans. In line with our hypothesis, we observed higher amounts of NPR-A in the brain tissue and lower levels of BNP in the CSF of AD patients. These findings further highlight that NP may be potential markers for AD. Given the widespread distribution of NP in different structures of the human brain, future research should determine the specific function of NP in the brain of humans, in health and disease. Finally, the inherent presence of NP in the brain and their easy availability in clinical practice are among the key factors that can accelerate the utility of NP as disease modifying agents in cognitive impairment and AD.

## Author Contributions

SM designed the experiments, performed the immunohisto-chemistry and western blot experiments, analyzed and interpreted the data, and drafted and revised the manuscript. MB designed the experiments, interpreted the immunohistochemistry and western blot data, and critically reviewed the manuscript. IvdV analyzed the immunohistochemistry data, collected the CSF data, and drafted and revised the manuscript. AM analyzed and interpreted the gene expression data, and critically reviewed the manuscript. WvR-M interpreted the immunohistochemistry and western blot data, and critically reviewed the manuscript. NF performed the ELISA measurements in CSF, and drafted and critically reviewed the manuscript. SY performed the ELISA measurements in CSF and critically reviewed the manuscript. BS, DvH, and LvdW conceived and designed the study, interpreted the data, and critically reviewed the manuscript.

## Conflict of Interest Statement

MB was employed by Percuros BV, Leiden, Netherlands. The remaining authors declare that the research was conducted in the absence of any commercial or financial relationships that could be construed as a potential conflict of interest.

## Footnotes

1 http://brainscope.nl/brainscope

2 http://human.brain-map.org/static/download

## References

[B1] Apostolova L. G. Thompson P. M. (2008). Mapping progressive brain structural changes in early Alzheimer’s disease and mild cognitive impairment. *Neuropsychologia* 46 1597–1612. 10.1016/j.neuropsychologia.2007.10.026 18395760 PMC2713100

[B2] Bohara M. Kambe Y. Nagayama T. Tokimura H. Arita K. Miyata A. (2014). C-type natriuretic peptide modulates permeability of the blood-brain barrier. *J. Cereb. Blood Flow Metab.* 34 589–596. 10.1038/jcbfm.2013.234 24398935 PMC3982079

[B3] Cao L.-H. Yang X.-L. (2008). Natriuretic peptides and their receptors in the central nervous system. *Prog. Neurobiol.* 84 234–248. 10.1016/j.pneurobio.2007.12.003 18215455

[B4] de Bold A. J. Borenstein H. B. Veress A. T. Sonnenberg H. (1981). A rapid and potent natriuretic response to intravenous injection of atrial myocardial extract in rats. *Life Sci.* 28 89–94. 10.1016/0024-3205(81)90370-2 7219045

[B5] Decker J. M. Wojtowicz A. M. Bartsch J. C. Liotta A. Braunewell K. H. Heinemann U. (2010). C-type natriuretic peptide modulates bidirectional plasticity in hippocampal area CA1 in vitro. *Neuroscience* 169 8–22. 10.1016/j.neuroscience.2010.04.064 20438814

[B6] Decker J. M. Wojtowicz A. M. Ul Haq R. Braunewell K. H. Heinemann U. Behrens C. J. (2009). C-type natriuretic peptide decreases hippocampal network oscillations in adult rats in vitro. *Neuroscience* 164 1764–1775. 10.1016/j.neuroscience.2009.09.036 19778593

[B7] Du A. T. Schuff N. Kramer J. H. Rosen H. J. Gorno-Tempini M. L. Rankin K. (2007). Different regional patterns of cortical thinning in Alzheimer’s disease and frontotemporal dementia. *Brain* 130(Pt 4), 1159–1166. 10.1093/brain/awm016 17353226 PMC1853284

[B8] Ermisch A. Ruhle H. J. Kretzschmar R. Baethmann A. (1991). On the blood-brain barrier to peptides: specific binding of atrial natriuretic peptide in vivo and in vitro. *Brain Res.* 554 209–216. 10.1016/0006-8993(91)90191-W 1657288

[B9] Gelfand R. A. Frank H. J. Levin E. Pedram A. (1991). Brain and atrial natriuretic peptides bind to common receptors in brain capillary endothelial cells. *Am. J. Physiol.* 261(2 Pt 1), E183–E189. 10.1152/ajpendo.1991.261.2.E183 1678581

[B10] Guo S. Goetze J. P. Jeppesen J. L. Burnett J. C. Olesen J. Jansen-Olesen I. (2015). Effect of natriuretic peptides on cerebral artery blood flow in healthy volunteers. *Peptides* 74 33–42. 10.1016/j.peptides.2015.09.008 26417835

[B11] Hawrylycz M. Miller J. A. Menon V. Feng D. Dolbeare T. Guillozet-Bongaarts A. L. (2015). Canonical genetic signatures of the adult human brain. *Nat. Neurosci.* 18 1832–1844. 10.1038/nn.4171 26571460 PMC4700510

[B12] Hawrylycz M. J. Lein E. S. Guillozet-Bongaarts A. L. Shen E. H. Ng L. Miller J. A. (2012). An anatomically comprehensive atlas of the adult human brain transcriptome. *Nature* 489 391–399. 10.1038/nature11405 22996553 PMC4243026

[B13] Hodes A. Lichtstein D. (2014). Natriuretic hormones in brain function. *Front. Endocrinol.* 5:201 10.3389/fendo.2014.00201 PMC4246887 25506340

[B14] Hu W. T. Holtzman D. M. Fagan A. M. Shaw L. M. Perrin R. Arnold S. E. (2012). Plasma multianalyte profiling in mild cognitive impairment and Alzheimer disease. *Neurology* 79 897–905. 10.1212/WNL.0b013e318266fa70 22855860 PMC3425844

[B15] Huisman S. M. H. van Lew B. Mahfouz A. Pezzotti N. Hollt T. Michielsen L. (2017). BrainScope: interactive visual exploration of the spatial and temporal human brain transcriptome. *Nucleic Acids Res.* 45:e83. 10.1093/nar/gkx046 28132031 PMC5449549

[B16] Iadecola C. (2004). Neurovascular regulation in the normal brain and in Alzheimer’s disease. *Nat. Rev. Neurosci.* 5 347–360. 10.1038/nrn1387 15100718

[B17] James M. L. Wang H. Venkatraman T. Song P. Lascola C. D. Laskowitz D. T. (2010). Brain natriuretic peptide improves long-term functional recovery after acute CNS injury in mice. *J. Neurotrauma* 27 217–228. 10.1089/neu.2009.1022 19803787

[B18] Kaneko T. Shirakami G. Nakao K. Nagata I. Nakagawa O. Hama N. (1993). C-type natriuretic peptide (CNP) is the major natriuretic peptide in human cerebrospinal fluid. *Brain Res.* 612 104–109. 10.1016/0006-8993(93)91649-D 8330189

[B19] Laakso M. P. Soininen H. Partanen K. Helkala E. L. Hartikainen P. Vainio P. (1995). Volumes of hippocampus, amygdala and frontal lobes in the MRI-based diagnosis of early Alzheimer’s disease: correlation with memory functions. *J. Neural Transm. Par Dis. Dement. Sect.* 9 73–86. 10.1007/BF02252964 7605591

[B20] Lehtovirta M. Laakso M. P. Soininen H. Helisalmi S. Mannermaa A. Helkala E. L. (1995). Volumes of hippocampus, amygdala and frontal lobe in Alzheimer patients with different apolipoprotein E genotypes. *Neuroscience* 67 65–72. 10.1016/0306-4522(95)00014-A 7477910

[B21] Mahinrad S. de Craen A. J. Yasar S. van Heemst D. Sabayan B. (2016). Natriuretic peptides in the central nervous system: novel targets for cognitive impairment. *Neurosci. Biobehav. Rev.* 68 148–156. 10.1016/j.neubiorev.2016.05.022 27229760

[B22] McKenzie J. C. (1992). Atrial natriuretic peptide-like immunoreactivity in astrocytes of parenchyma and glia limitans of the canine brain. *J. Histochem. Cytochem.* 40 1211–1222. 10.1177/40.8.1535643 1535643

[B23] McKenzie J. C. Berman N. E. Thomas C. R. Young J. K. Compton L. Y. Cothran L. N. (1994). Atrial natriuretic peptide-like (ANP-LIR) and ANP prohormone immunoreactive astrocytes and neurons of human cerebral cortex. *Glia* 12 228–243. 10.1002/glia.440120308 7851990

[B24] Moriyama N. Taniguchi M. Miyano K. Miyoshi M. Watanabe T. (2006). ANP inhibits LPS-induced stimulation of rat microglial cells by suppressing NF-kappaB and AP-1 activations. *Biochem. Biophys. Res. Commun.* 350 322–328. 10.1016/j.bbrc.2006.09.034 17010309

[B25] Ogawa Y. Nakao K. Nakagawa O. Komatsu Y. Hosoda K. Suga S. (1992). Human C-type natriuretic peptide. Characterization of the gene and peptide. *Hypertension* 19(6 Pt 2), 809–813. 1339402 10.1161/01.hyp.19.6.809

[B26] Pemberton C. J. Yandle T. G. Espiner E. A. (2002). Immunoreactive forms of natriuretic peptides in ovine brain: response to heart failure. *Peptides* 23 2235–2244. 10.1016/S0196-9781(02)00263-2 12535704

[B27] Potter L. R. Abbey-Hosch S. Dickey D. M. (2006). Natriuretic peptides, their receptors, and cyclic guanosine monophosphate-dependent signaling functions. *Endocr. Rev.* 27 47–72. 10.1210/er.2005-0014 16291870

[B28] Prado J. Baltrons M. A. Pifarré P. García A. (2010). Glial cells as sources and targets of natriuretic peptides. *Neurochem. Int.* 57 367–374. 10.1016/j.neuint.2010.03.004 20302900

[B29] Sabayan B. van Buchem M. A. de Craen A. J. Sigurdsson S. Zhang Q. Harris T. B. (2015). N-terminal pro-brain natriuretic peptide and abnormal brain aging: the AGES-Reykjavik study. *Neurology* 85 813–820. 10.1212/WNL.0000000000001885 26231259 PMC4553023

[B30] Suzuki T. Yamazaki T. Yazaki Y. (2001). The role of the natriuretic peptides in the cardiovascular system. *Cardiovasc. Res.* 51 489–494. 10.1016/S0008-6363(01)00238-3 11476739

[B31] Telegdy G. Adamik A. Glover V. (2000). The action of isatin (2,3-dioxoindole) an endogenous indole on brain natriuretic and C-type natriuretic peptide-induced facilitation of memory consolidation in passive-avoidance learning in rats. *Brain Res. Bull.* 53 367–370. 10.1016/S0361-9230(00)00359-2 11113594

[B32] van der Velpen I. F. Feleus S. Bertens A. S. Sabayan B. (2017). Hemodynamic and serum cardiac markers and risk of cognitive impairment and dementia. *Alzheimers Dement.* 13 441–453. 10.1016/j.jalz.2016.09.004 27770635

[B33] Wang T. J. Larson M. G. Levy D. Benjamin E. J. Leip E. P. Omland T. (2004). Plasma natriuretic peptide levels and the risk of cardiovascular events and death. *N. Engl. J. Med.* 350 655–663. 10.1056/NEJMoa031994 14960742

[B34] Wijsman L. W. Sabayan B. van Vliet P. Trompet S. de Ruijter W. Poortvliet R. K. (2014). N-terminal pro-brain natriuretic peptide and cognitive decline in older adults at high cardiovascular risk. *Ann. Neurol.* 76 213–222. 10.1002/ana.24203 24942833

