# Supplementary material for: Natriuretic Peptides in Post-mortem Brain Tissue and Cerebrospinal Fluid of Non-demented Humans and Alzheimer’s Disease Patients
Source: Front Neurosci. 2018 Nov 26;12:864. doi: 10.3389/fnins.2018.00864 (PMC6275179; doi:10.3389/fnins.2018.00864)
Supplement: Supplementary file 1 [file Data_Sheet_1.pdf]

SUPPLEMENTAL MATERIAL

Supplementary Table 1. List of antibodies used in this study

ID	Company	Description	Host	Antigen recognized	AR
ab14356	Abcam	Anti-NPRA	Rb (pc)	aa 294-308 of h-NPRA	+
ab55724	Abcam	Anti-NPRB	Ms (mc)	aa 131-231 of h-NPRB	+
ab37617	Abcam	Anti-NPRC	Rb (pc)	aa 67-97 (N terminal) h-NPRC	+
ab19646	Abcam	Anti-BNP	Rb (pc)	h-BNP	+
ab91250	Abcam	Anti-ANP	Rb (pc)	aa 30-56 of h-ANP	–
HPA035362	Sigma	Anti-NPPC	Rb (pc)	aa 29-126 of h-CNP	+

Abbreviations: Rb: rabbit; Ms: mouse; pc: polyclonal; mc: monoclonal; AR: antigen retrieval; h: human and aa: amino acid.

Supplementary Table 2. Semi-quantitative comparison of staining signals in different cell structures between AD patients and controls

	ANP	BNP	CNP	NPR-A	NPR-B	NPR-C
% +scores in AD patients / % +scores in controls, n=23 (13 controls and 10 AD patients)						
Neurons	100/100	100/100	100/100	100/100	100/100	100/100
Astrocyte-like shapes	100/100	0/0	10/0	70/23.1	60/31	60/23
			(p=0.244)	(**p=0.024)**	(p=0.161)	(p=0.072)
Leptomeningeal vessels	100/100	100/100	0/0	100/100	100/100	100/90
						(p=0.305)

Each subject was scored as present (+) or absent (-) of signal for each protein. The percentage of +/- scorings were used to compare between AD patients and controls. P-values were calculated using chi-squared test.

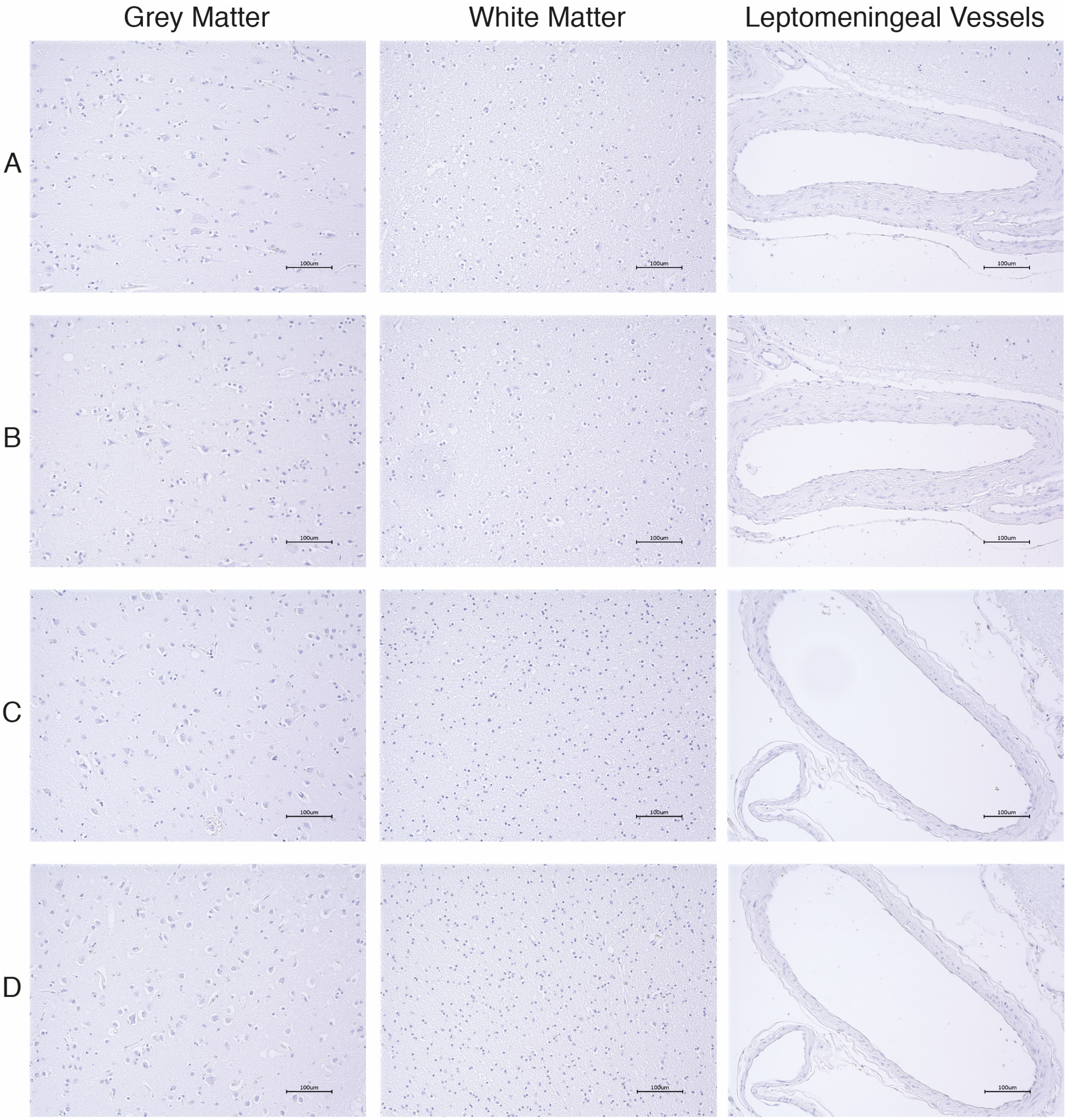

Supplementary Figure 1. Negative controls treated only with secondary antibodies

A) Frontal cortex of a control subject incubated only with secondary goat anti rabbit antibody and B) secondary rabbit anti mouse antibody. C) Frontal cortex of an AD patient incubated only with secondary goat anti rabbit antibody and D) secondary rabbit anti mouse antibody

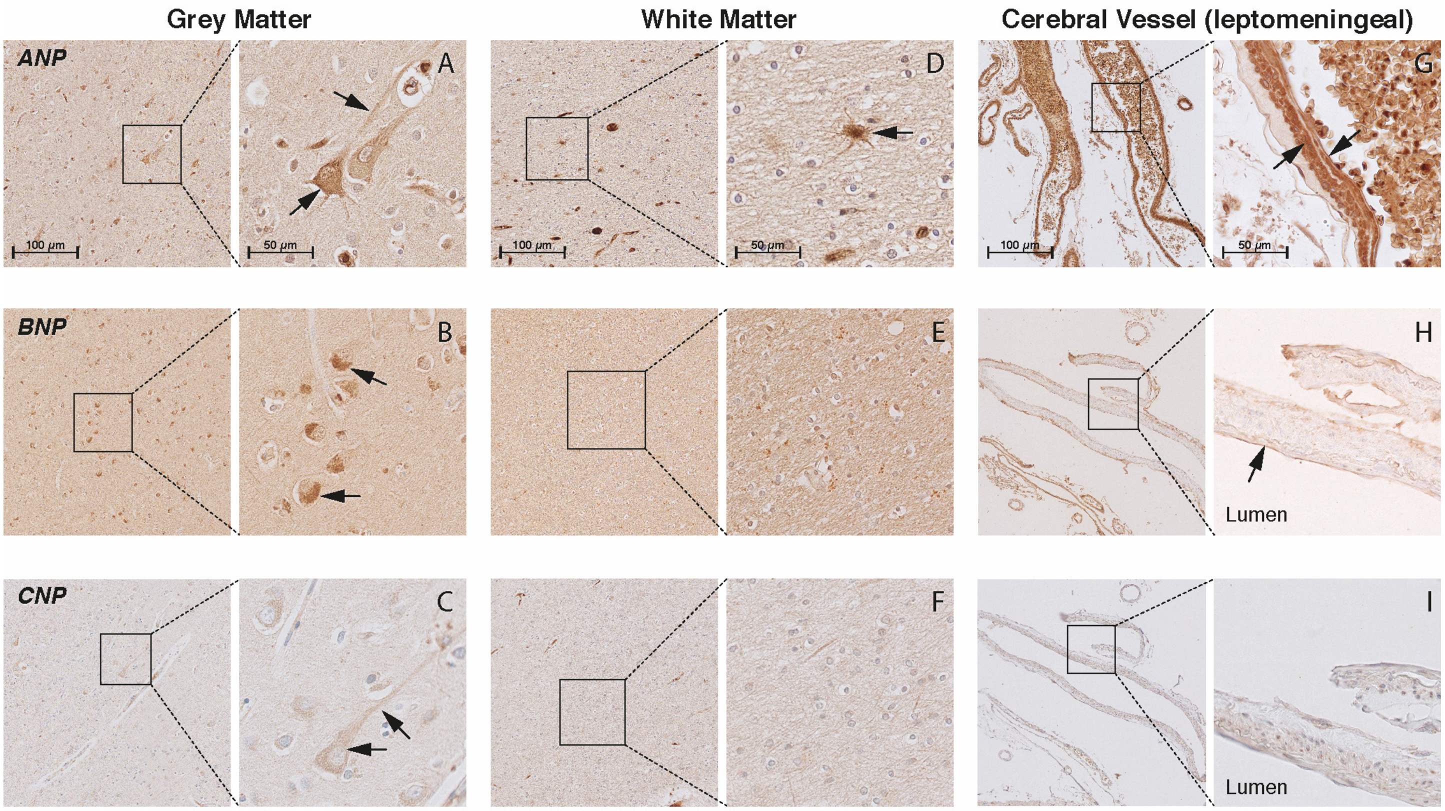

Supplementary Figure 2. Localization of ANP, BNP and CNP in the frontal lobe of AD subjects

A, B and C) ANP, BNP and CNP-positive neurons. Arrows point to cytoplasm (ANP and CNP), neuronal processes (ANP, and CNP) and Nissle bodies (BNP); D) ANP-positive astrocyte-like cells in the white matter; E and F) negative BNP and CNP staining in the white matter; G) ANP-positive endothelium and smooth muscles; H) BNP-positive endothelium and I) negative CNP staining in the leptomeningeal vessels.

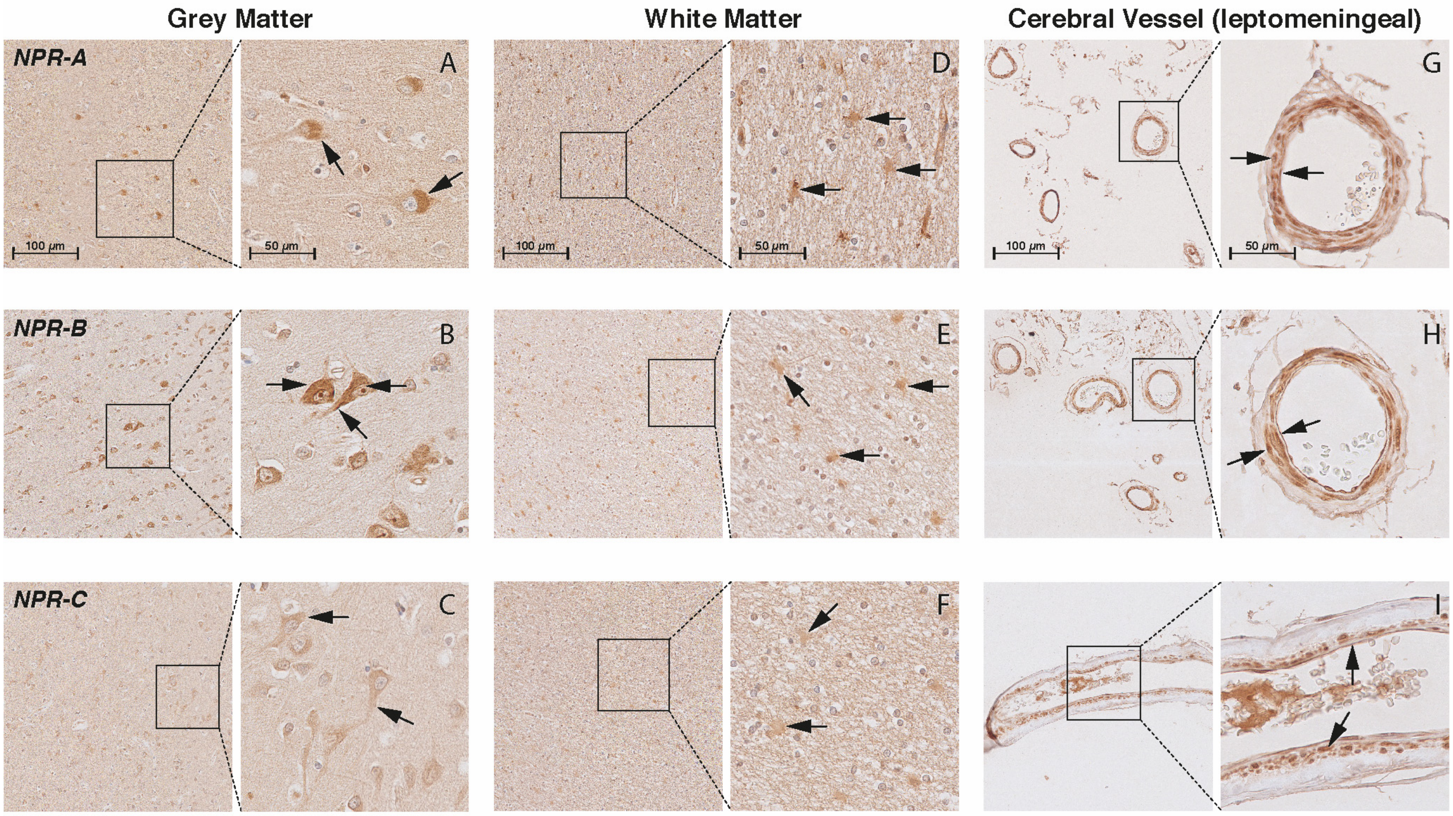

Supplementary Figure 3. Localization of NPR-A, NPR-B and NPR-C in the frontal lobe of AD subjects
A, B and C) NPR-A, NPR-B and NPR-C-positive neurons. Arrows point to cytoplasm (all NPR), neuronal processes (all NPR) and Nissl bodies (NPR-A); D, E and F) NPR-A, NPR-B and NPR-C-positive astrocyte-like cells in the white matter; G, H and I) NPR-A, NPR-B and NPR-C-positive endothelium and smooth muscles in leptomeningeal vessels

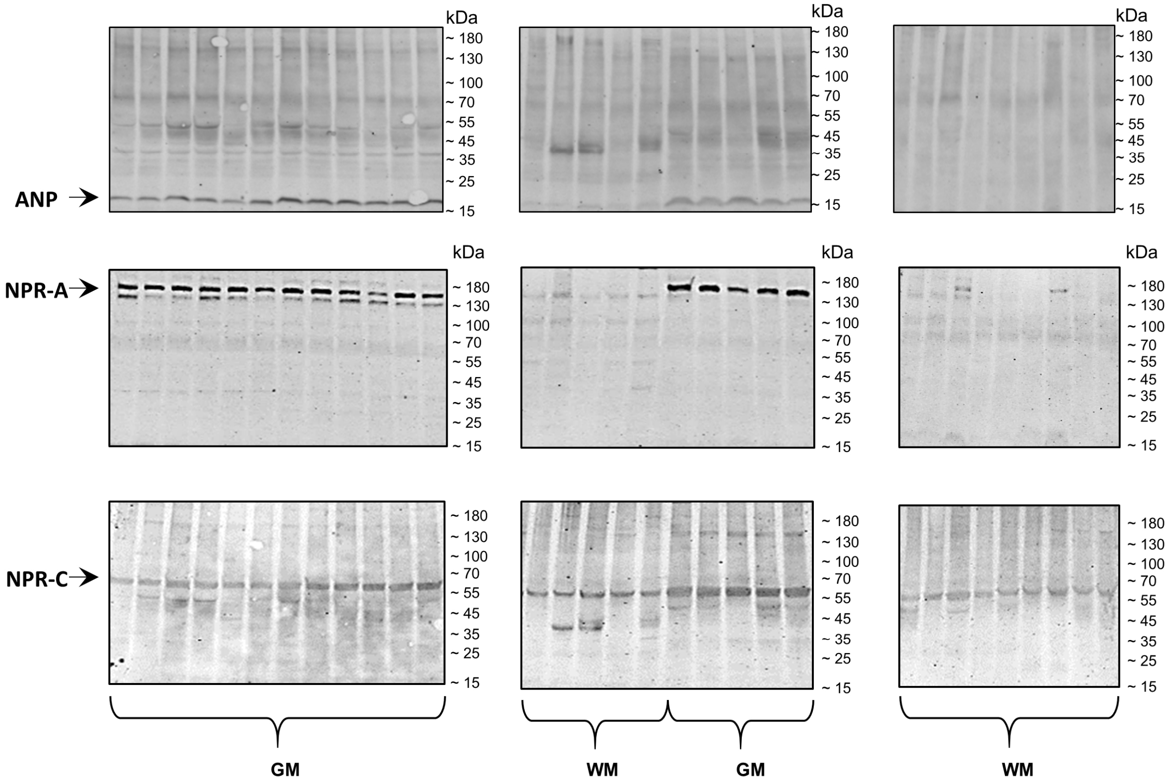

Supplementary Figure 4. Western blots of ANP, NPR-A and NPR-C. Each subject has one band corresponding to grey matter and one band corresponding to white matter. All images were scanned using the Image Studio software with the same resolution and image quality. Abbreviations: ANP: atrial natriuretic peptide; NPR-A: natriuretic peptide receptor A; NPR-C: natriuretic peptide receptor C; GM: grey matter and WM: white matter.
